# RNA modifications in pulmonary diseases

**DOI:** 10.1002/mco2.546

**Published:** 2024-05-03

**Authors:** Weiwei Qian, Lvying Yang, Tianlong Li, Wanlin Li, Jian Zhou, Shenglong Xie

**Affiliations:** ^1^ Emergency Department of Emergency Medicine Laboratory of Emergency Medicine, West China Hospital, And Disaster Medical, Sichuan University Chengdu Sichuan China; ^2^ Emergency Department Shangjinnanfu Hospital, West China Hospital, Sichuan University Chengdu Sichuan China; ^3^ The Department of Respiratory and Critical Care Medicine The First Veterans Hospital of Sichuan Province Chengdu Sichuan China; ^4^ Department of Critical Care Medicine Sichuan Provincial People's Hospital University of Electronic Science and Technology of China Chengdu Sichuan China; ^5^ National Clinical Research Center for Infectious Disease, Shenzhen Third People's Hospital Shenzhen Guangdong China; ^6^ Guangdong Key Laboratory for Biomedical Measurements and Ultrasound Imaging, National‐Regional Key Technology Engineering Laboratory for Medical Ultrasound, School of Biomedical Engineering, Shenzhen University Medical School Shenzhen China; ^7^ Department of Immunology International Cancer Center, Shenzhen University Health Science Center Shenzhen Guangdong China; ^8^ Department of Thoracic Surgery Sichuan Provincial People's Hospital, University of Electronic Science and Technology of China Chengdu Sichuan China

**Keywords:** pulmonary diseases, functions of RNA modifications, RNA modification database, RNA modifications

## Abstract

Threatening public health, pulmonary disease (PD) encompasses diverse lung injuries like chronic obstructive PD, pulmonary fibrosis, asthma, pulmonary infections due to pathogen invasion, and fatal lung cancer. The crucial involvement of RNA epigenetic modifications in PD pathogenesis is underscored by robust evidence. These modifications not only shape cell fates but also finely modulate the expression of genes linked to disease progression, suggesting their utility as biomarkers and targets for therapeutic strategies. The critical RNA modifications implicated in PDs are summarized in this review, including N^6^‐methylation of adenosine, N^1^‐methylation of adenosine, 5‐methylcytosine, pseudouridine (5‐ribosyl uracil), 7‐methylguanosine, and adenosine to inosine editing, along with relevant regulatory mechanisms. By shedding light on the pathology of PDs, these summaries could spur the identification of new biomarkers and therapeutic strategies, ultimately paving the way for early PD diagnosis and treatment innovation.

## Graphical abstract

This review presents the crucial involvement of RNA epigenetic modifications (m^6^A, m^1^A, m^5^C, Ψ, m^7^G, and A‐to‐I editing) in pulmonary diseases, spanning chronic conditions to lung cancer. Focusing on their impact on disease regulation as promising biomarkers and therapeutic targets. Additionally, essential databases and tools are summarized, fostering insights for clinical research of pulmonary disease to improve diagnosis and treatment. m^6^A, N^6^‐methylation of adenosine; m^1^A, N^1^‐methylation of adenosine; m^5^C, 5‐methylcytosine; Ψ, pseudouridine; m^7^G, 7‐methylguanosine; A‐to‐I, adenosine to inosine; COPD, chronic obstructive pulmonary disease; IPF, interstitial lung disease, idiopathic pulmonary fibrosis; ARDS, acute respiratory distress syndrome; PTB, pulmonary tuberculosis; RMBase, RNA Modification Base Database; NGS, next‐generation sequencing; LC–MS, liquid chromatography mass spectrometer; TLC, thin layer chromatography; RIP, RNA immunoprecipitation.

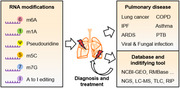

## 1 INTRODUCTION

Chronic obstructive pulmonary disease (COPD), pneumonia, asthma, interstitial lung disease, idiopathic pulmonary fibrosis (IPF), acute respiratory distress syndrome (ARDS), pulmonary tuberculosis (PTB), and fatal lung cancer are all part of the spectrum of pulmonary diseases (PDs) affecting the lungs and airways.^1^, ^2^ Various harmful factors, including inhalation of particulate matter or gases, allergen stimulation, invasion by pathogens, dysregulation of the immune system, abnormal cell apoptosis, oxidative stress, or aberrant inflammation induced by other diseases, contribute to the onset and progression of PDs.^3^, ^4^, ^5^ PD represents a significant proportion of global deaths, with the COVID‐19 outbreak intensifying this burden on a global scale.^6^, ^7^, ^8^ Committed to reducing the burden of respiratory diseases on patients and society, the Global Alliance against Chronic Respiratory Diseases (GRD) has been established by the World Health Organization (WHO). Despite substantial strides in understanding the genome and signaling pathways associated with PDs, as well as the advent of new treatments, the mortality rate persists with little alteration. The significant diversity found in the majority of PD cases underscores the ongoing necessity for novel, validated methods in both diagnosis and treatment.

**FIGURE 1:**
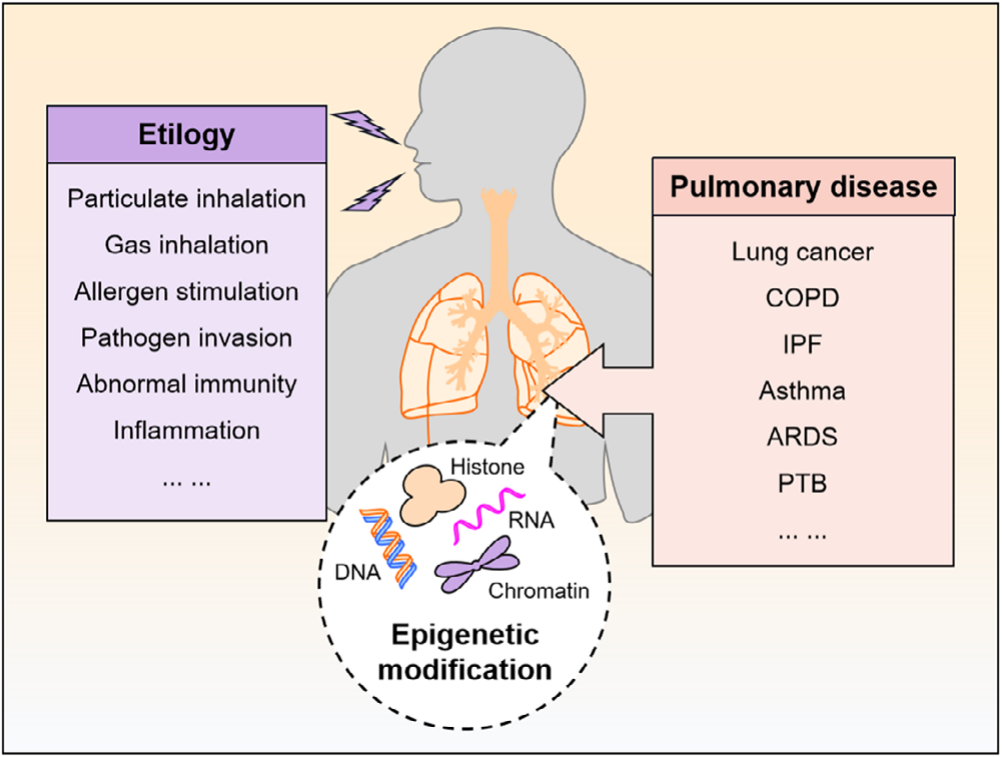
Epigenetic modifications in pulmonary diseases. Pulmonary diseases are short for a variety of complex lung injuries, including lung cancer, chronic obstructive pulmonary disease (COPD), idiopathic pulmonary fibrosis (IPF), asthma, acute respiratory distress syndrome (ARDS), and pulmonary tuberculosis (PTB), which can be triggered by multiples of factors like particulate and gas inhalation, allergen stimulation, pathogen invasion (viruses, bacteria and fungi), abnormal immunity, and inflammation. Epigenetic modifications containing DNA, RNA, chromatin, and histone are involved in the occurrence and progression of pulmonary diseases, and are also the long‐term research focus of diagnosis and targeted therapy of pulmonary diseases.

Epigenetics, a domain within molecular biology, concerns itself with the examination of heritable changes in gene expression devoid of alterations in DNA sequences. DNA and RNA methylation, chromatin remodeling, noncoding RNA, and histone modifications constitute the primary mechanisms of epigenetic modifications.^9^ Investigation into modifications spanning the entire genome has yielded insights into how genetic changes in diverse regions are linked to the complexity of respiratory diseases.^10^ New avenues for biomarker discovery and therapeutic targeting in PDs have opened up with the evolution of RNA epigenetics.^11^ Incorporating advancements in gene dynamic regulation has revolutionized the utilization of epigenetics in both diagnosing and treating PDs, offering new avenues for exploration.^12^ In PD pathogenesis, a complex interplay of epigenetic modifications extensively regulates cell phenotypes and molecular mechanisms.^13^ Epigenetics and the application of targeted therapies based on epigenetic mechanisms have seen a growing utilization in clinical settings. This trend significantly enhances the prospects for early detection, management, and prevention of PDs.^14^ A plethora of studies have consolidated findings on the interplay between RNA modifications and PDs, with specific attention directed toward lung cancer.^15^, ^16^, ^17^, ^18^ Moreover, considerable attention has been devoted to investigating the link between RNA modifications and PDs, beyond lung cancer. Hence, uncovering the intricacies of the RNA modification process is pivotal in elucidating the pathogenesis and progression of PDs (Figure 1).

Through this review, we seek to elucidate the intricate relationship between RNA modifications and PDs, while also highlighting key RNA modification databases and functional tools. By doing so, we aim to offer valuable references and perspectives for comprehensively understanding the etiology and pathology of PDs.

## 2 RNA MODIFICATIONS IN PDs

### 2.1 RNA modification pathways and enzymes

**FIGURE 2:**
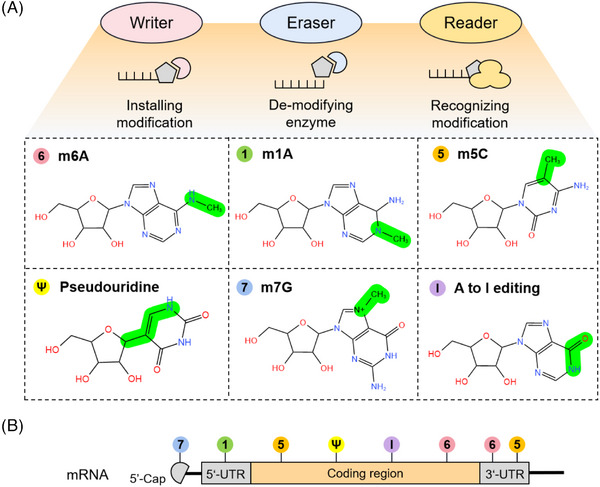
Several critical RNA modification types. (A) The common RNA modification types include N^6^‐methylation of adenosine (m^6^A), N1‐methylation of adenosine (m^1^A), 5‐methylcytosine (m^5^C), pseudouridine (5‐ribosyl uracil, Ψ), 7‐methylguanosine (m^7^G), and adenosine to inosine (A‐to‐I) editing, which are regulated by three RNA modifying enzymes. The “writer” enzyme is responsible for installing modifications while the “eraser” enzyme removes it. The “reader” protein binds and recognizes RNA modification marks on target RNAs to impact RNA fate. (B) Various RNA modifications are enriched in different regions of mRNA. RNA modifications regulate the stability, translation, and localization of disease‐related mRNA through a “writing‐erasing‐reading” mechanism, thereby controlling the progression of disease.

The past ten years have been marked by significant advancements in understanding RNA epigenetics, emphasizing its crucial role in fundamental biological processes pertinent to PDs. For instance, in the process of transcription, splicing, and translation, RNA modifications not only participate in protein synthesis and regulate gene expression as messenger RNA (mRNA), transfer RNA (tRNA), and ribosomal RNA (rRNA), but also act directly on gene expression in the form of multiple types of noncoding RNA (ncRNA), such as microRNA (miRNA), small nuclear RNA (snRNA), and long ncRNA (lncRNA).^19^, ^20^, ^21^, ^22^, ^23^ With PD included, the count of recognized RNA modifications exceeds 170, all instrumental in numerous disease‐associated biological processes.^24^, ^25^ RNA modifications, which entail editing standard AUCG bases, impact base pairing, secondary structure, and the RNA's ability to directly interface with proteins. Further, RNA processing, localization, translation, and degradation are all subject to modulation by these chemical changes, thereby governing gene expression.^26^ RNA modifications are a process that can be both reversible and irreversible,^27^, ^28^ and are dynamically regulated by three specialized protein tools known as “writers,” “erasers,” and “readers.” Completed by various methyltransferases (MTases), writers perform the installation of RNA modifications on RNA substrates. Demethylases, acting as “erasers,” facilitate the reversible removal of select RNA modifications, while “readers,” RNA modification binding proteins, identify these alterations. This dynamic interplay governs RNA stability and modulates key biological functions such as splicing, translation, and localization post modified RNA recruitment.^15^ Certainly, an association exists between dysregulated m^6^A modification regulators and multiple cellular processes as well as diseases. For example, across a spectrum of cancer cells, irregular RNA modifications are prevalent, exerting effects on oncogene expression and fostering both tumor growth and metastasis.^29^ Not only does the dysregulation of RNA modification regulators affect the pluripotency and differentiation of embryonic stem cells^30^ but it is also implicated in neurodevelopmental disorders and neurodegenerative diseases.^31^ RNA modifications, notably m^6^A, are instrumental in governing immune responses, as adjustments in RNA modification regulators within immune cells can impact the expression of genes associated with immune function, inflammation, and responses to pathogens.^25^ RNA modification regulators, when deregulated in heart cells, also play a role in the development of cardiovascular diseases.^32^ Key RNA modifications pertinent to PDs include N^6^‐methylation of adenosine (m^6^A), N^1^‐methylation of adenosine (m^1^A), 5‐methylcytosine (m^5^C), pseudouridine (5‐ribosyl uracil, Ψ), 7‐methylguanosine (m^7^G), and adenosine to inosine (A‐to‐I) editing (Figure 2A).^33^ By categorizing RNA and applying distinct treatments, modifications influence its trajectory in processes such as cellular differentiation, embryonic development, and responses to stress. Provided below is a summary of notable RNA modifications and their regulatory components associated with PDs (Table 1).

**TABLE 1:** Several critical RNA modifications involved in PDs and their regulatory molecules.

Modifications	Structure^a^	Regulatory molecule^b^		
		Writer	Eraser	Reader
m^6^A	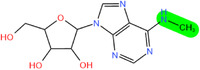	METTL3, METTL14, METTL16, WTAP, RBM15, RBM15B, ZC3H13, VIRMA	FTO, ALKBH1, 3, 5	YTHDF1‐3, YTHDC1, 2 IGF2BP
m^1^A	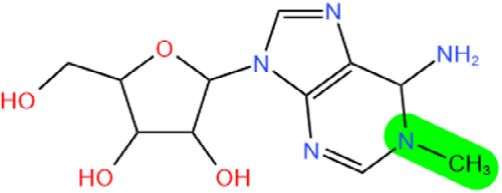	TRMT10C, TRMT61B	ALKBH1, ALKBH3	YTHDF1‐3, YTHDC1
m^5^C	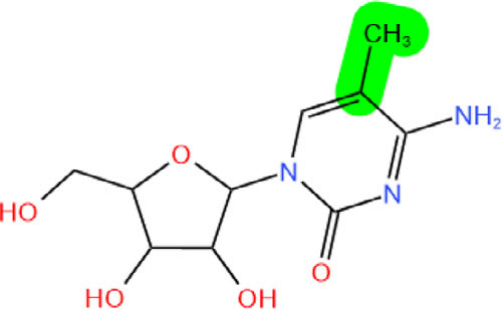	NSUN1‐7, DNMT2	TET1−3, ALKBH1	ALYREF, YBX1, YTHDF2
Ψ	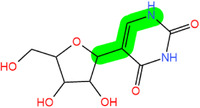	DKC1, PUS1, PUS3, TRUB1 (PUS4), TRUB2, RPUSD1−4, PUS6, PUS7, PUS7L, PUS10	/	/
m^7^G	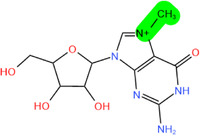	RNMT, METTL1/WDR4, WBSCR22	/	/
A‐to‐I editing	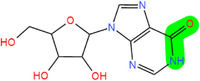	ADAT2/3, ADAR1‐3	/	/

Abbreviation: PDs, pulmonary diseases.

^a^ “
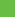
” in “Structure” indicates the RNA methylation site.

^b^ “/” in “Regulatory molecule” indicates regulatory molecule of specific RNA modifications is recently not found.

### 2.2 Key RNA modifications related to PDs

#### 2.2.1 m^6^A modifications

Posttranscriptional RNA modification involves the addition of the m^6^A methylation group, which exhibits a significant presence adjacent to the protein‐coding region, mRNA stop codon, and the 3′ untranslated region (3′‐UTR) (Figure 2B).^34^, ^35^ The abundant methylation modification m^6^A, widely distributed in eukaryotic cells, serves to regulate gene expression posttranscriptionally through chemical modifications, while preserving the mRNA sequence.^36^, ^37^ Across the spectrum of the RNA lifecycle, encompassing RNA splicing, nuclear export, translation, expression, and metabolism, m^6^A methylation holds significant importance.^38^, ^39^ The central component of the “writer” complex responsible for RNA methylation consists primarily of the METTL3/METTL14 heterodimer. This complex exhibits selective recognition of particular sites within RNA sequences, facilitating m6A methylation reactions.^40^ METTL3, the pioneering m^6^A methyltransferase, is renowned for its high degree of conservation among diverse organisms.^41^ Within cytoplasmic domains, METTL3 functions in translation initiation irrespective of its methyltransferase capabilities.^42^ METTL14 enhances the catalytic effect of m^6^A RNA methylation or serves as an RNA‐binding scaffold to promote substrate recognition.^43^ Within the realm of hypothetical methyltransferases, METTL16 stands out as a key catalyst for the methylation of U6 snRNA and ncRNA m^6^A.^44^ Methylation sites differ between the two “writers.” In the context of methylation preferences, the METTL3/14 complex predominantly targets the RRACH sequence motif (with R = A or G; H = A, U, or C) for m^6^A methylation, whereas METTL16 specifically favors the UACGAGAA sequence within the protrusion of the “duckbill” stem‐loop RNA, particularly methylating the A4 position.^45^, ^46^ Wilms’ tumor 1‐associating protein (WTAP), RNA‐binding motif protein 15 (RBM15), RBM15B, zinc finger CCCH domain‐containing protein 13 (ZC3H13), and vir‐like m^6^A methyltransferase associated protein (VIRMA) act as regulatory subunits, augmenting the catalytic ability of m^6^A.^47^, ^48^, ^49^ In a mechanism reliant on Fe^2+^—and α‐ketoglutarate, the “eraser” complex, constituted by fat mass and obesity‐associated protein (FTO) and alpha‐ketoglutarate‐dependent dioxygenase alkB homolog 5 (ALKBH5), eliminates m^6^A methylation.^28^, ^50^ Mainly responsible for tRNA demethylation, ALKBH3 is another member of the ALKBH family.^51^ Initially uncovered as the “reader,” the YT521‐B homology domain (YTHD) family consists of two subfamilies: DF (YTHDF1, 2, 3) and DC (YTHDC1, 2).^52^ The binding of YTHDF1 and eukaryotic translation initiation factor 3 (EIF3) can improve the translation efficiency of m^6^A‐modified RNA targets.^53^ Recruiting the CCR4–NOT complex, YTHDF2 is involved in the degradation process of m^6^A‐modified transcripts.^54^ Intriguingly, THDF3 shows synergistic effects with the diverse regulatory functions of these two proteins.^55^, ^56^ By recruiting and regulating pre‐mRNA splicing factors, YTHDC1 facilitates mRNA splicing by targeting the binding region of its target mRNA.^57^ Not only does YTHDC2 stimulate mRNA translation, but it also oversees the stability of m^6^A‐modified mRNA.^58^, ^59^ With their recognized function as m^6^A “readers,” the insulin‐like growth factor‐2 mRNA‐binding protein (IGF2BP) family members drive the recruitment of RNA stabilizers, fostering mRNA stability and translation and consequently impacting gene expression.^60^

#### 2.2.2 m^1^A modifications

Homologous to m^6^A, m^1^A represents a less common RNA methylation marker, its activity being controlled by a variety of methyltransferase complexes and demethylases.^61^ Mainly involved in modifying tRNA and rRNA, m^1^A modification of mRNA is concentrated in its 5′‐UTR (Figure 2B).^62^, ^63^ Catalyzing distinct sites, TRMT10C and TRMT61B facilitate the m1A modification at m^1^A9 and m^1^A58 on human mitochondrial tRNA, respectively.^64^, ^65^ Within vertebrate mitochondrial ribosomes, the TRMT61B‐driven m^1^A modification at position 947 of 16S rRNA remains constant, promoting the stability of the ribosomal framework.^66^ The ribosomal peptide transfer center within nuclear‐encoded large rRNA features a conserved m^1^A site that is shared between budding yeast and humans.^67^, ^68^ Human nuclear protein nucleomethylin, alternatively recognized as RRP8, catalyzes the m^1^A modification at position 1322 of 28S rRNA.^69^ Not only is m^6^A reversible, but m1A has also been identified as a second reversible RNA modification, indicating that ALKB family proteins including ALKBH1 and ALKBH3 can remove m^1^A methylation from tRNA and mRNA.^70^, ^71^ YTHDF1‐3 and YTHDC1, members of the YTH protein family, share the capacity to bind to the m^1^A marker as potential readers, unlike YTHDC2.^72^

#### 2.2.3 m^5^C modifications

Near the translation initiation site or 3′‐UTR is where m^5^C methylation exhibits a preference (Figure 2B). Alongside mRNA, rRNA, tRNA, enhancer RNA (eRNA), and ncRNA, other RNA species undergo m^5^C methylation,^73^ a process pivotal for regulating RNA output, ribosome assembly, translation, and expression. m^5^C modifications execute different functions in different RNA subtypes. For example, m^5^C is related to RNA structure and stability in tRNA and is also necessary for translation accuracy.^73^ Lack of m^5^C methylation at position 2278 of yeast 25S rRNA leads to temporary rRNA presence and encourages stop codon read‐through.^74^ Enzymes from the NOL1/NOP2/SUN domain (NSUN) family, comprising seven members (NSUN1‐7), are responsible for catalyzing m^5^C methylation in eukaryotes.^75^ NSUN1 and NSUN5 modify 28S rRNA, while NSUN3 and NSUN4 modify mitochondrial tRNA and rRNA, respectively. Cytoplasmic tRNA, mRNA, and several ncRNAs, such as vault RNA (vtRNA), eRNA, and lncRNA, are subject to modification by NSUN2, while NSUN7 focuses on eRNA. In addition, DNA methyltransferase 2 (DNMT2) functions as a “writer,” engaging in modifications of cytoplasmic tRNAs.^76^ ALYREF, an RNA methyltransferase, is recognized as the pioneering “reader” of m^5^C modification. ALYREF‐mediated mRNA binding fosters the accumulation of m^5^C near the translation initiation codon and in the CG sequence, which in turn facilitates mRNA nuclear export.^77^, ^78^ Located in the cytoplasm, the “reader” YBX1 recruits ELAV‐like RNA binding protein 1 (ELAVL1) to enhance the stability of m^5^C‐modified mRNA.^79^ In its role as an m^6^A “reader” protein, YTHDF2 exhibits the ability to directly associate with m^5^C in RNA, thus controlling the distribution of m^5^C in both coding and noncoding RNAs and affecting rRNA maturation.^80^ m^5^C modification “erasers” include ten‐eleven translocation proteins (TET1‐3) and α‐ketoglutarate‐dependent dioxygenase ABH1 (ALKBH1). The activity of TET involves oxidizing m^5^C in RNA to 5‐hydroxymethylcytidine (hm5C).^81^, ^82^ ALKBH1 induces the conversion of m^5^C to hm5C and 5‐formylcytosine at position 34 of both cytoplasmic and mitochondrial tRNA.^83^, ^84^

#### 2.2.4 Ψ modifications

Synthesized by pseudouridine synthase (PUS), pseudouridine (Ψ) is a posttranscriptional RNA modification found abundantly in tRNA and rRNA, and it is conserved across prokaryotic and eukaryotic organisms.^85^ At the structural level, Ψ influences RNA by enhancing stability and changing translation efficiency.^86^ Essential for ribosome assembly, Ψ modifications in the rRNA domain also alter the functional traits of ribosomes.^87^ Moreover, both snRNA and mRNA (Figure 2B) demonstrate Ψ modifications, where the modification in snRNA influences mRNA splicing efficiency.^88^ mRNA Ψ modifications are contingent upon particular RNA structural properties that are considered necessary and sufficient.^89^ Dyskerin 1 (DKC1), one of the thirteen identified “writers” of Ψ in humans, acts as a catalytic subunit assembled by small nuclear ribonucleoprotein (snRNP) complexes, enabling the catalysis of rRNA Ψ modifications.^90^ Comprising the additional 12 “writers” are RNA‐dependent PUS enzymes, namely PUS1, PUS3, TRUB1 (also referred to as PUS4), TRUB2, RPUSD1‐4, PUS6, PUS7, PUS7L, and PUS10. The enzymes demonstrate precise localization within cells and target specific RNA sequences.^91^, ^92^, ^93^ Nevertheless, the quest for Ψ “erasers” or “readers” remains inconclusive. The lack of recognized Ψ “erasers” may be due to the formation of relatively inert C−C bonds between ribose and base groups, resulting in an irreversible Ψ process.^94^

#### 2.2.5 m^7^G modifications

Positioned at the 5′ cap of eukaryotic mRNA, m^7^G is a positively charged modification that has been evolutionarily conserved, with roles in controlling mRNA export, translation, and splicing (Figure 2B).^95^, ^96^ Internally, m^7^G is detected at position 1581 of tRNA, position 46 of tRNA, and within mature human miRNA sequences.^97^, ^98^ RNA guanine‐7 methyltransferase (RNMT), along with the METTL1‐WD repeat domain 4 (WDR4) complex and the Williams‐Beuren syndrome chromosomal region 22 protein (WBSCR22), is involved in the methylation of m^7^G, acting as “writers.”^99^, ^100^ The METTL1 and WDR4 complexes demonstrate m^7^G methyltransferase activity toward both tRNA and mRNA.^101^ WBSCR22 can interact with tRNA methyltransferase activator subunit 11‑2 (TRMT112) to specifically methylate m^7^G in 18S rRNA.^102^

#### 2.2.6 A‐to‐I editing

Across mammalian mRNA, ncRNA, and even viral RNA (Figure 2B), A‐to‐I editing stands out as a special irreversible posttranscriptional modification.^103^, ^104^, ^105^ A‐to‐I editing alters the secondary structure of RNA by converting adenosine to inosine via deamination,^106^, ^107^ thus affecting target miRNA through splicing site regulation, and modifying the amino acid sequence of proteins.^108^ The process of converting adenosine to inosine in tRNA involves adenosine deaminase acting on tRNA (ADAT). ADAT2 and ADAT3 collaborate as a heterodimer to mediate the conversion of adenosine to inosine at the wobble position (position 34) in eukaryotic tRNAs.^109^, ^110^ Moreover, the adenosine deaminase acting on the dsRNA (ADAR) family is implicated in the editing process of dsRNA.^108^ However, ADAR3 could compete for binding to dsRNA, resulting in decreased enzyme activity and editing capacity.^111^, ^112^ No evidence exists at present to suggest that “erasers” and “readers” further regulate A‐to‐I editing.

## 3 FUNCTIONS OF RNA MODIFICATIONS IN PDs

### 3.1 Lung cancer

#### 3.1.1 Insights of RNA modifications for lung cancer therapy

Respiratory diseases, particularly lung cancer, have been frequently linked to RNA modifications in numerous reports.^16^, ^17^, ^18^ Lung cancer, as of 2020, emerged as the second most common malignancy globally, representing 11.4% of all new cancer diagnoses and standing as the primary contributor to cancer‐related deaths, claiming 1.8 million lives annually (18%).^113^ It is well accepted that one of the significant hallmarks of cancer is the aberrant expression of genes, which is linked to the accumulation of genetic and epigenetic changes driving the occurrence and progression of human cancers. In some cases, cancer cells manifest epigenetic and signaling similarities akin to those found in stem cells, giving rise to a stem cell‐like phenotype.^114^ Accumulated epigenetic changes within the respiratory epithelium drive the upregulation of oncogenes and the repression of tumor suppressor genes.^115^ Thus, the strong correlation between abnormal RNA methylation patterns and cancer predisposition is evident. Investigating the epigenetic mechanisms of RNA may yield new insights into biomarkers and therapeutic targets for lung cancer, thereby enabling advancements in early detection, treatment, and prognosis monitoring.

The past few years have marked an unparalleled advancement in diagnostic modalities and targeted therapeutics for lung cancer.^116^ However, targeted therapy may not be advantageous for all lung cancer patients, given that drug resistance inevitably arises, hampering therapeutic effectiveness and ultimately leading to unfavorable survival outcomes. Reports indicate that extended use of therapeutic medications, such as tyrosine kinase inhibitors, tends to lead to acquired resistance and disease progression in cancer patients. Associations between alterations in RNA epigenetic modifications and poor efficacy, as well as dismal prognosis, have been observed in cancer patients.^117^ In addition to their role in cancer cell functions such as proliferation, metastasis, metabolism, and regulation,^118^, ^119^ RNA modifications also have implications for drug resistance.^94^ Thus, the reversal of epigenetic events might emerge as a novel approach for personalized interventions in oncology.

#### 3.1.2 Mechanisms of m^6^A modifications in lung cancer

Despite previous explicit reviews discussing different regulators associated with m^6^A modification in lung cancer,^15^, ^16^, ^17^, ^18^ our review provides a systematic overview. Linked to the activation of lung cancer‐related signaling pathways are the various signaling pathways associated with m^6^A methylation, the most abundant RNA modification in eukaryotic cells. m^6^A modifications control key epigenetic transcriptional events in lung cancer such as cell differentiation and cancer metabolism.^39^, ^120^ Cancer cell self‐renewal and fate are influenced by the m^6^A modification of mRNA, as evidenced by research.^121^ METTL3, identified as a significant m^6^A “writer,” demonstrates elevated expression in human lung cancer tissues, driving the methylation process of mRNA and ncRNA and ultimately enhancing tumor progression.^122^ In addition, METTL3 is related to the tumor stage of primary lung adenocarcinoma (LUAD).^123^ The promotion of cancer cell apoptosis and the regulation of p53 signal transduction are observed upon knockdown of METTL3, indicating its critical role in cancer cell survival.^123^, ^124^ Correspondingly, findings suggest that METTL3 can be subject to modification by a small ubiquitin‐like modifier (SUMO), resulting in the inhibition of m^6^A methyltransferase activity and subsequent reduction in mRNA m^6^A levels. The SUMOylation of METTL3 significantly promotes the growth of human non‐small cell lung cancer (NSCLC).^125^ However, METTL3's impact on tumor progression may occur independently of m^6^A. For example, irrespective of its catalytic activity, upregulation of METTL3 drives the growth and invasion of LUAD cells by binding to eIF3 and enhancing the translation of target transcripts such as epidermal growth factor receptor (EGFR) and transcriptional co‐activator with PDZ‐binding motif (TAZ).^42^ Accelerating ribosomal circulation, METTL3 facilitates the translation of the oncogene Bromodomain‐containing protein 4 (BRD4) by creating an mRNA loop with eIF3h, thereby contributing to the enhanced translation of oncogenes.^123^ This unravels that METTL3's involvement extends beyond catalyzing m^6^A to participating in the postmethylation regulation of target mRNA, thus playing a role in cancer development. In the realm of tumor metabolism, METTL3 induces m^6^A methylation of lncRNA ABHD11‐AS1, which enhances the stability of its transcript, resulting in increased expression. This, in turn, promotes NSCLC cell proliferation, triggers the Warburg effect, and disrupts normal tumor glucose metabolism.^126^

The epithelial–mesenchymal transition (EMT) is additionally highlighted as a fundamental driving force behind malignant tumors.^127^ METTL3's involvement in the transmission and metastasis of lung cancer cells includes mediating EMT through its m^6^A catalytic activity.^127^ An increase in m^6^A modifications and METTL3 expression occurs in A549 and LC‐2/ad lung cancer cells upon induction of EMT by transforming growth factor‐β (TGF‐β). Reduction of m^6^A modification of JUNB, a critical transcription factor governing EMT, occurs upon METTL3 knockout. Consequently, mRNA stability decreases, impeding TGF‐β‐induced cell morphological transformation, and suppressing the EMT phenotype.^128^ The recruitment of m^6^A modification by IGF2BP3 on minichromosome maintenance complex component 5 (MCM5) mRNA can trigger EMT and enhance the adaptability of LUAD cells via m^6^A‐dependent overactivation of the Notch signal, ultimately driving tumor metastasis.^129^ The reduction in METTL3 expression mediated by miR‐338‐5p disrupts nuclear oncogene c‐Myc‐mediated m^6^A modifications, leading to decreased expression of c‐Myc and consequent suppression of the proliferation, invasion, and migration of lung cancer cells.^130^ Enrichment of m^6^A RNA modifications in circulating tumor cells (CTCs) of lung cancer patients^131^ suggests the potential utility of METTL3 as a biomarker for lung cancer diagnosis, prognosis, and treatment. Heightened levels of m^6^A RNA methylation may be implicated in monitoring and thwarting tumor metastasis.

Alterations in the expression of m^6^A RNA methyltransferases and demethylases in lung cancer can impair RNA functionality. Increasing the stability of mRNA transcripts through demethylation boosts protein expression, consequently promoting the proliferation of lung cancer cells. Independent regulation coexists with mutual regulation in the regulatory mechanisms of m^6^A demethylases and recruitment protein YTHD family in lung cancer. The significant elevation of ALKBH1 expression, facilitated by the functional upregulation of crucial residues Y184, H231, D233, H287, R338, and R344 in demethylation, promotes lung cancer cell migration and invasion.^132^ Lung squamous cell carcinoma (LUSC) demonstrates heightened expression of FTO, which drives the expression of the oncogene MZF1 by decreasing m^6^A levels and mRNA stability within the MZF1 mRNA transcript. Consequently, this process promotes carcinogenesis.^133^ Inducing hypoxia‐mediated tumor proliferation, ALKBH5 downregulates m^6^A modifications on Forkhead box M1 (FOXM1) mRNA and enhances FOXM1 protein expression, consequently fostering LUAD progression.^134^ Enhancing cancer progression in lung cancer cells, YTHDF2 directly binds to the m^6^A modification site within the 3′‐UTR of 6PGD, promoting the translation of 6‐phosphogluconate dehydrogenase (6PGD) mRNA and augmenting the pentose phosphate pathway (PPP).^135^ Within NSCLC, ALKBH5 exerts control over the miR‐107–LATS2 signaling cascade, dampening the activity of YAP, a significant driver of solid tumors in humans. This modulation involves decreasing m^6^A modifications and YTHDF‐mediated YAP expression, ultimately restraining cancer cell proliferation and metastasis, presenting a potential targeted therapy approach for lung cancer.^136^ In LUAD, low expression of FTO is associated with the activation of the Wnt/β‐catenin signaling pathway. YTHDF1, in conjunction with elevated c‐Myc m^6^A levels, contributes to tumor glycolysis and growth in LUAD.^137^

**FIGURE 3:**
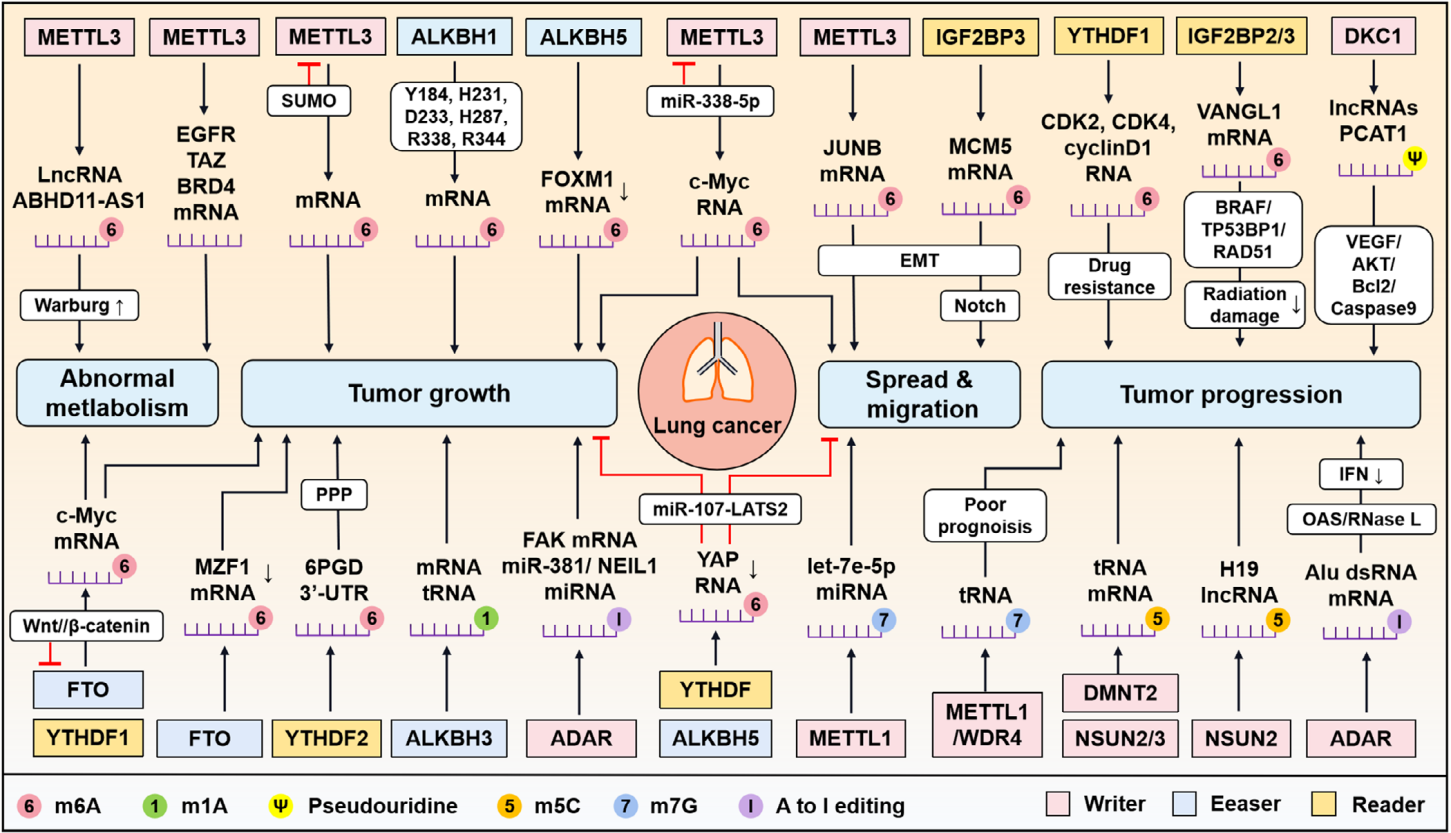
The mechanisms of RNA modifications in lung cancer. The critical RNA modifications associated with the occurrence and progression of lung cancer include m^6^A, m1A, Ψ, m^5^C, m7G, and A‐to‐I editing. In short, these modifications influence metabolism, growth, metastasis, and migration of tumor, as well as participate in the progression of lung cancer. First, writer METTL3‐mediated lncRNA ABHD11‐AS1 m^6^A methylation leads to abnormal glucose metabolism. Decreased m^6^A methylation of MZF1 mRNA by FTO facilitates tumor growth. Inhibition of FTO by Wnt/β‐catenin enhances the m^6^A modification of c‐Myc mRNA after binding to YTHDF1, and then promotes glycolysis and tumor growth. Second, SUMO1‐modified METTL3 as well as Y184, H231, D233, H287, R338, and R344‐activated ALKBH1 can promote mRNA m^6^A modifications; ALKBH5 decreases the m^6^A modification of FOXM1 mRNA; all facilitate tumor growth. The YTHDF2‐mediated 6PGD 3′‐UTR m^6^A modification promotes tumor growth through PPP pathway. ALKBH3‐induced mRNA and tRNA m^1^A modifications, ADAR‐edited FAK mRNA and miR‐381/NEIL1 miRNA also enhance tumor growth. Next, m6A‐modified c‐Myc mRNA enhances the growth, metastasis, and migration of cancer cells through METTL3, which can be canceled by miR‐338‐5p. ALKBH5 decreases m^6^A modifications and YTHDF mediates YAP expression, therefore inhibiting tumor growth and migration through miR‐107–LATS2 signaling. IGF2BP3 induces EMT to promote tumor metastasis and migration by Notch signaling after recognizing the m^6^A modification of MCM5 mRNA. Knockdown of METTL3 regulates JUNB mRNA and reverses EMT phenotype. METTL1‐accumulated m^7^G methylation on let‐7e‐5p miRNA inhibits tumor migration. Finally, YHDF1‐regulated drug resistance and IGF2BP2/3‐mediated radiation damage influence cancer progression. Ψ methyltransferase DKC1 accelerates cancer progression through VEGF/AKT/Bcl2/Caspase9 pathway. Overexpression of METTL1/WDR4 is associated with poor prognosis. NSUN2/3 and DMNT2‐induced abnormal m^5^C modifications promote tumor occurrence and development. A‐to‐I editing influences tumor progression by disturbing IFN‐dependent immune responses.

The standard procedures for cancer treatment in clinical practice continue to involve chemotherapy and radiotherapy. Increasing attention has been directed toward the biological significance of m^6^A modifications and their potential regulatory mechanisms in impacting chemotherapy response and radiotherapy sensitivity. NSCLC responds to hypoxic microenvironments by downregulating YTHDF1, thereby inducing cisplatin resistance through the modulation of CDK2, CDK4, and cyclin D1 translation.^138^ The stability and expression of VANGL1 mRNA postradiation are affected in LUAD as a consequence of the deletion of IGF2BP2/3 in m^6^A modifications. The knockout of VANGL1 may interfere with the BRAF–TP53BP1–RAD51 cascade, resulting in DNA damage and amplifying the adverse impact of radiation on LUAD (Figure 3).^139^ Briefly, serving as novel biomarkers, reversible m^6^A markers present on transcripts demonstrate a growing potential for advancing the early diagnosis and treatment of lung cancer.

#### 3.1.3 Other RNA modifications in lung cancer

Highly expressed in NSCLC, m1A demethylase ALKBH3 demonstrates a significant correlation with poor prognosis. Cell cycle arrest, induction of cell senescence, and inhibition of cell growth in LUAD cell lines are observed upon knockdown of ALKBH3.^140^ ALKBH3‐mediated demethylation of tRNA m1A in lung cancer cells, alongside mRNA modifications, leads to the accumulation of tRNA‐derived small RNAs and tRNA‐derived fragments. This accumulation enhances ribosome assembly, elevates translation rates, and mitigates cell apoptosis.^141^ tRNA m^5^C is also significantly upregulated in CTCs of lung cancer patients, and NSUN2 is highly expressed in a wide range of human malignancies.^131^, ^142^ NSUN2‐mediated aberrant m^5^C modification of lncRNA H19 drives the occurrence and evolution of hepatocellular carcinoma.^143^ NSUN3 and DNMT2 tRNA's m^5^C modification is implicated in tumor development as well.^144^, ^145^ Despite efforts, the exact workings of m^5^C modifications in cancers remain unclear, underscoring the necessity for further exploration to delineate the potential role of m^5^C in the progression of lung cancer. The mutation of dyskeratosis congenita 1 (DKC1), an enzyme reliant on small nucleolar RNA (snoRNA) for Ψ modification, can trigger the onset of various cancers, including liver cancer and prostate cancer.^146^, ^147^ Lung cancer presents heightened expression levels of lncRNA PCAT1, which interacts with DKC1 to influence NSCLC cell proliferation, invasion, and apoptosis via the VEGF/AKT/Bcl2/Caspase9 pathway, ultimately driving tumor progression.^148^ Nevertheless, the regulatory mechanisms governing DKC1 and other Ψ modifications require further exploration. METTL1 serves as a “writer” of m^7^G, facilitating the deposition of m^7^A onto let‐7e‐5p miRNA and promoting its processing into mature let‐7miRNA. Through disruption of inhibitory secondary structures within the primary miRNA transcript, this mechanism curtails tumor development by suppressing genes associated with metastasis.^98^ However, overexpression of METTL1/WDR4 is also indicative of poor prognosis in lung cancer patients. METTL1 knockdown hampers tRNA modifications and lowers mRNA translation, consequently diminishing the invasion and carcinogenicity of lung cancer cells.^149^ Consequently, the advantages of METTL1 in lung cancer have yet to be fully elucidated. Variations in specific malignant tumors make both A‐to‐I editing and ADAR enzymes viable candidates for specific biomarkers.^103^ Enhancing focal adhesion kinase (FAK) mRNA stability involves the main mediator of A‐to‐I editing, ADAR, binding to the FAK transcript, and editing specific intron sites. This mechanism enriches FAK gene expression, consequently promoting tumor recurrence. Repressing cancer cell migration/invasion and reversing the carcinogenic process can be achieved by knocking out ADAR in LUAD cells.^150^ Reductions in interferon (IFN) and other inflammatory mediators bolster the immune evasion of tumors, shaping immune regulation. ADAR1 overexpression typically curtails the dsRNAs generated by retrotransposon Alu repetitive sequences, thus mitigating their capacity to induce IFN immune responses and facilitating tumor progression.^151^ The presence of ribonuclease L (RNase L) is essential to prevent cell death triggered by ADAR1 deficiency in human LUAD cell lines. Under specific conditions, IFN‐mediated activation of the oligoadenylate synthetase/Rnase L system might act as a mediator for antiviral reactions.^152^ Furthermore, ADAR1 depletion or the use of ADAR1 small molecule inhibitors presents a potential therapeutic avenue for cancer patients, augmenting cellular sensitivity to immune checkpoint blockade.^105^, ^153^ Another carcinogenic mechanism of ADAR1 in lung cancer is the regulation of miRNA processing. Heightened ADAR1 expression induces an elevated editing frequency in target transcripts of tumor suppressor factors like miR‐381 and NEIL1, contributing to substantial growth of NSCLC cells.^154^ The alteration in miRNA editing levels signifies a promising biomarker candidate for LUAD (Figure 3).^155^

### 3.2 Chronic obstructive pulmonary disease

In addition, the impact of RNA modifications on COPD has also been explored. Arising from exposure to inhaled noxious particles such as tobacco smoke and pollutants, COPD is a chronic progressive disease. Characteristics commonly observed in COPD patients comprise mucosal ciliary dysfunction, small airway obstruction, pulmonary inflammation, and emphysema.^156^ Approximately 650,000 individuals suffer from COPD each year, primarily in low‐income nations, solidifying its position as the third leading cause of mortality worldwide.^157^ Epigenetic changes are among the multiple pathogenic factors implicated in COPD.^158^ Regulatory functions of epigenetic modifications in COPD's onset, progression, and clinical prognosis have also been demonstrated.^159^, ^160^ Abnormal changes in miRNA activity have been proven to accelerate the onset of COPD.^161^ Pulmonary inflammatory responses in COPD patients are attributed to dysregulated miRNA, impacting disease advancement.^162^ Consequently, RNA epigenetic changes offer biomarkers to diagnose COPD or determine disease status.^163^

RNA modifications in COPD have garnered less research interest compared to DNA posttranscriptional modifications.^164^ Existing research primarily targets m^6^A RNA modifications, unveiling m^6^A modifications in 4500 genes in the lung tissues of mice with stable COPD. Around 2000 m^6^A methylation peaks are observed in acute COPD, with these differential methylation patterns notably linked to immune function and inflammation.^160^ The occurrence of COPD shows a significant correlation with mRNA expression levels of RNA methylation regulatory factors, as unveiled by bioinformatics analysis of 24 common m^6^A RNA methylation regulatory factors via the STRING database.^165^ COPD patients present upregulated IGF2BP3 expression while downregulated FTO, YTHDC1, and YTHDC2 expressions compared with healthy controls. These regulatory factors indirectly interact with several key COPD‐related genes such as BCL2A1, GPX2, AKR1B10, ALDH3A1, CABYR, CYP4F3, EGF, UCHL1, CYP1A1, CYP1B1, and MUCL1. In COPD‐related gene expression, writers METTL3 and YTHDC2 demonstrate a positive correlation, whereas the eraser FTO exhibits a negative correlation, implying a role for m^6^A modifications in COPD.^165^ In addition, continual low levels of METTL3 expression in adulthood may hinder m^6^A modifications, subsequently impacting lung development. Impaired lung function leads to hypoxia, triggering pulmonary arterial hypertension, a frequent complication seen in COPD.^166^ In terms of inflammation induction, lncRNA small nuclear RNA host gene 4 (SNHG4) exacerbates lipopolysaccharide (LPS)‐induced pulmonary inflammatory responses by inhibiting the m^6^A modification of STAT2 mRNA mediated by METTL3.^167^ Further research is needed to demonstrate whether the abnormal interactions of these m^6^A RNA methylation regulatory factors lead to the occurrence of COPD.

**FIGURE 4:**
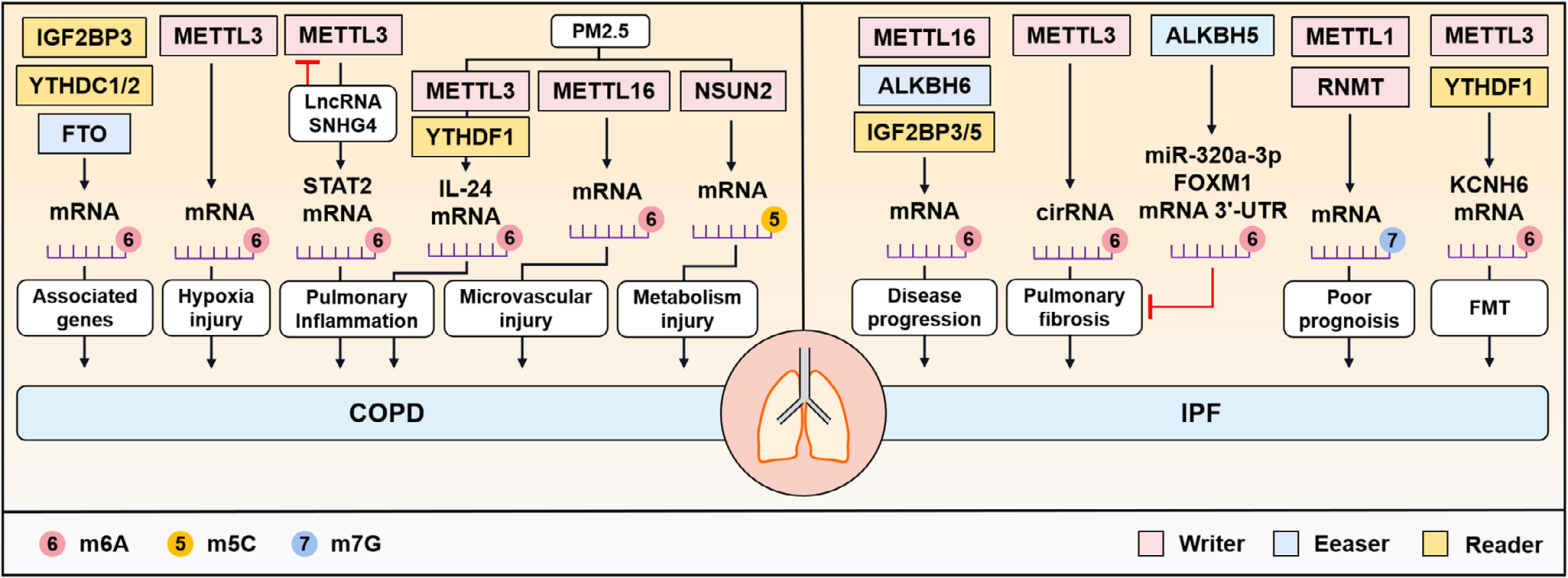
The mechanisms of RNA modifications in CPOD and IPF. RNA modifications regulate the development of other PDs including COPD and IPF. Of which, m^6^A is the most studied modification. For COPD, IGF2BP3, YTHDC1, YTHDC2, and FTO interact with disease‐associated genes. mRNA m6A methylation mediated by METTL3 induces hypoxia injury and pulmonary inflammation, leading to outcome of COPD. PM2.5‐caused COPD involves inflammatory response promoted by METTL3, microvascular injury induced by the METTL16‐mediated m^6^A modification, and metabolism injury caused by NSUN2‐mediated m^5^C methylation. For IPF, METTL16, ALKBH6, IGFBP3, and IGFBP5 promote disease progression after m^6^A methylation. METTL3‐induced pulmonary fibrosis can be reversed by m^6^A demethylase ALKBH5 after targeting miR‐320a‐3p and FOXM1 mRNA 3′‐UTR. METTL1 and RNMT‐regulated m^7^G modifications is related to poor prognosis. The m^6^A modification of KCNH6 promotes FMT process in a YTHDF1‐dependent manner, and thus causing IPF. Silencing METTL3 can hinder FMT process.

Fine particulate matter (PM2.5) inhalation in excess is a key factor in COPD etiology, with exposure to PM2.5 precipitating the expression of m^6^A regulatory factors and the advancement of PDs.^17^ Increased expression of METTL3 following PM2.5 exposure is intricately tied to pulmonary inflammation and mucus production, contributing to heightened IL‐24 m^6^A modifications and mRNA stability. In addition, PM2.5‐induced upregulation of YTHDF1 additionally boosts the translation efficiency of IL‐24 mRNA. Therefore, m^6^A regulators mediate inflammatory damage through METTL3/YTHDF1.^168^ In the induction of microvascular injury by PM2.5, METTL16 m^6^A modifications are additionally implicated, thereby fostering the progression of COPD.^169^ In addition to m^6^A, the NSUN2‐mediated mRNA m^5^C modification might adversely affect normal lung metabolic activity by upregulating gene expression in the lungs of mice subjected to PM2.5.^170^ Collectively, elucidating the link between RNA modifications and diseases, new therapeutic strategies for COPD are illuminated, along with a deeper understanding of the regulatory mechanisms of other RNA modifications in COPD pathogenesis (Figure 4).

### 3.3 Idiopathic pulmonary fibrosis

IPF is also a chronic and fatal lung disease, which is also associated with RNA modifications to a certain extent. In IPF, there is an ongoing and irreversible accumulation of collagen in the lung parenchyma, which disrupts gas exchange over time and culminates in mortality.^171^ The unclear etiology of IPF continues to pose challenges in developing therapeutic methods that effectively prevent or reverse fibrosis, despite ongoing advancements in treatment strategies.^172^ PM2.5 inhalation elevates IPF risk and hastens lung function deterioration in patients.^171^ PM2.5‐induced pulmonary fibrosis tissues exhibit an abnormal increase in mRNA m^5^C modifications,^170^ indicating the implication of epigenetic mechanisms in pulmonary fibrosis pathogenesis.

Significant overexpression of m^6^A regulators IGFBP3, IGFBP5, and ALKBH6 characterizes IPF patients, with a positive association between elevated METTL16 levels and ALKBH6, highlighting the pivotal role of m^6^A modification regulators as IPF biomarkers.^173^ The analysis of sequencing data from IPF patients in the GEO database highlights METTL3, a regulatory factor involved in m^6^A methylation, as a promising candidate for predicting risk or assessing prognosis in IPF.^174^ METTL3‐mediated circRNA m^6^A modifications have been confirmed to correlate with SiO_2_‐induced lung fibroblast activation and migration, as well as pulmonary fibrosis outcomes.^175^ Similarly, the m^6^A demethylase ALKBH5 promotes lung fibroblast activation and silica‐induced pulmonary fibrosis by targeting miR‐320a‐3p and/or FOXM1 mRNA 3′‐UTR, while ALKBH5 knockdown reverses the fibrosis process..^176^ High METTL1 expression correlates with adverse prognosis in IPF patients, allowing for the classification of IPF into two molecular subtypes based on the expressions of the m^7^G regulatory genes (METTL1 and RNMT). Subtype 2 patients face a poorer prognosis than subtype 1 patients, highlighting the importance of m^7^G in prognostic prediction and early diagnosis of IPF.^177^

Myofibroblast abnormal aggregation stands out as a key pathology in IPF, primarily arising from resident fibroblasts through the fibroblast to myofibroblast transition (FMT) pathway.^178^ m^6^A modifications exhibit increased expression in bleomycin (BLM)‐induced in vitro and in vivo models of pulmonary fibrosis, as well as in lung samples from individuals with IPF. Mechanistically, m^6^A modifications regulate the expression of KCNH6 in a YTHDF1‐dependent manner, thereby participating in the FMT process. Silencing of METTL3 can abate m^6^A modifications and hinder the FMT process both in vivo and in vitro.^179^ There exist multiple mechanisms for RNA epigenetic changes in IPF, and targeting RNA modification regulators may contribute to the early diagnosis of IPF and the future development of immunotherapy strategies for IPF (Figure 4).

### 3.4 m^6^A modifications in other PDs

Actually, the pathogenesis of PDs is complex, and the types of PDs are also diverse. In addition to common lung cancer, COPD, and IPF, m^6^A modifications are also prominent in various other rare types of PDs. Summarized in Table 2 are the mechanisms of m^6^A modifications in different lung injuries, including the three types of PDs mentioned earlier (Table 2).

**TABLE 2:** Summary of m^6^A modifications in pulmonary diseases.

Disease	Mechanisms	Outcomes	References
Lung cancer	SUMO‐modified METTL3 promotes mRNA m^6^A methylation in NSCLC	Tumor growth	^125^
	METTL3‐mediated LncRNA ABHD11‐AS1 m^6^A methylation increases transcript stability in NSCLC	Abnormal metabolism	^126^
	METTL3 promotes JUNB modification and mRNA stability	Tumor spread and migration	^128^
	IGF2BP3 recognizes MCM5 m^6^A modification and promotes EMT by Notch signaling	Tumor spread and migration	^129^
	METTL3 promotes c‐Myc mRNA modification, which can be inhibited by miR‐338‐5p	Tumor growth Tumor spread and migration	^130^
	Y184, H231, D233, H287, R338, and R344 activated ALKBH1 promotes mRNA m^6^A modification	Tumor growth	^132^
	FTO decreases m^6^A levels of MZF1 transcript and stability of mRNA in LUSC	Tumor growth	^133^
	ALKBH5 inhibits FOXM1 mRNA m^6^A modification in LUAD	Tumor growth	^134^
	YTHDF2 binds to 6PGD 3′‐UTR m^6^A modification sites and facilitates PPP pathway	Tumor growth	^135^
	ALKBH5 decreases YTHDF mediated YAP modification that activates miR‐107‐LATS2 signaling in NSCLC	Inhibit tumor growth Inhibit tumor spread and migration	^136^
	Decrease expression of FTO by Wnt/β‐catenin signaling and combination of YTHDF1 on c‐Myc mRNA promote m6A methylation in LUAD	Abnormal metabolism Tumor growth	^137^
	YHDF1 regulated CDK2, CDK4, and cyclin D1 modification promotes drug resistance in NSCLC	Promote tumor progression	^138^
	IGF2BP2/3 mediated VANGL1 modification reduces radiation damage by BRAF/TP53BP1/RAD51 cascade in LUAD	Promote tumor progression	^139^
COPD	IGF2BP3, YTHDC1, YTHDC2, and FTO interact with disease associated genes	Participate in COPD	^165^
	Low expression of METTL3 induces m^6^A modification has adverse effects on lung development	Hypoxia injury	^166^
	METTL3 promotes STAT2 m^6^A modification, which can be inhibited by SNHG4	Pulmonary inflammation	^167^
	METTL3 and YTHDF1 enhance IL‐24 m^6^A modification and stability of mRNA	Pulmonary inflammation	^168^
	METTL16 increases m^6^A modification of mRNA	Microvascular injury	^169^
IPF	METTL16, ALKBH6, IGFBP3, and IGFBP5 promote m^6^A methylation	Promote disease progression	^173^
	METTL3 increase m^6^A modification of circRNA	Pulmonary fibrosis	^175^
	ALKBH5 recognizes m^6^A modifications of miR‐320a‐3p and FOXM1 mRNA 3′‐UTR	Inhibit pulmonary fibrosis	^176^
	METTL3 increases m^6^A modificationYTHDF1 regulates KCNH6 mRNA expression	Participate in FMT	^179^
ARDS	Regulatory molecule expressed dynamically during the course of the disease	Pulmonary inflammation	^180^
Asthma	YTHDF3 influence asthma‐related eosinophil	Promote disease progression	^181^, ^182^
	FTO promotes the mRNA stability of the ciliary transcription factor FOXJ1	Inhibit asthma progress	^183^
Viral infection	Increase of METTL3/METTL14 and YTHDF1, 2, 3; decrease of ALKBH5/FTO	Promote RSV replication	^185^
	METTL3 regulates the splicing efficiency of proteins encoded by fiber genes	Promote late AdV replication	^186^
	Over expression of METTL3 and YTHDF2	Promote IAV replication	^187^
	Over expression of writer protein	Promote HMPV replication	^188^
	Decreased METTL3 located m^6^A modification inhibits the stress response, which can be accompanied by XOP1 inhibition	Promote SARS‐CoV‐2 infection	^189^
Fungal infection	Decrease expression of YTHDF1, 2, 3, YTHDC1 and YTHDC2 in PTB	Participate in MT infection	^190^

Abbreviations: ARDS, acute respiratory distress syndrome; COPD, chronic obstructive pulmonary disease; IPF, idiopathic pulmonary fibrosis.

In LPS‐induced ARDS mouse tissues, m^6^A methylation‐related factors exhibit dynamic changes with the prolongation of infection time, and the overall m^6^A RNA methylation level significantly increases, indicating that inflammatory factor regulation and ARDS development may be influenced by m^6^A methylation induced by LPS. m^6^A modifications may be a favorable candidate for ARDS therapy.^180^ m^6^A methylation is also implicated in the emergence and evolution of asthma, with the most crucial regulatory factor being the YTHDF3 enriched at exon 3′‐UTR, which has an impact on asthma related eosinophils.^181^, ^182^ FTO regulates motile ciliogenesis. The deficiency of FTO caused the destabilization of FOXJ1 mRNA, which governs the expression of a critical transcription factor essential for ciliary function, consequently displaying potent asthma‐like features.^183^ Therefore, intervening in m^6^A modifications may guide therapeutic strategies for various PDs.

Pathogen invasion can bring a huge burden to the lungs. The m^6^A modification of the viral genome is crucial for its lifecycle.^184^ Respiratory viruses are common pathogens. Human respiratory syncytial virus (RSV) is susceptible to modification by m^6^A. m^6^A methyltransferase (METTL3 and/or METTL14) promotes the replication and gene expression of RSV, while the “reader” proteins (YTHDF1, 2, 3) further promote the production of offspring viruses. m^6^A demethylase (ALKBH5 and/or FTO) has the opposite effect.^185^ The transcripts of adenovirus (AdV) at the early and late stages contain METTL3‐dependent m^6^A modifications. Knockout of METTL3 can affect the late transcript of the virus by reducing the splicing efficiency of proteins encoding fiber genes in the later stage of infection.^186^ Influenza A virus (IAV) expresses RNA with m^6^A modifications, and the m^6^A residue in its transcript enhances viral gene expression. YTHDF2 overexpression further promotes the production of viral particles. Knockout of METTL3 yields a decrement in viral gene expression, replication, and pathogenicity.^187^ Similarly, m^6^A methylation of human metapneumovirus (HMPV) RNA also facilitates HMPV replication and gene expression, and the protein and RNA levels of the virus are remarkably upregulated due to overexpression of the “writer” protein.^188^ Additionally, the previously prevalent severe acute respiratory syndrome coronavirus 2 (SARS‐CoV‐2) impairs stress response and disrupts cellular gene expression via an m^6^A‐dependent pathway. Interestingly, m^6^A is lost in SARS‐CoV‐2‐infected cells and enriched in viral RNA, which is related to the different localization of METTL3 in the cytoplasm. Inhibiting the output protein XOPX1 can restore the localization of METTL3 and m^6^A modifications on cell RNA, further relieving stress particle damage and reducing in vitro viral infection.^189^ In addition to viral infection, m^6^A “readers” YTHDF1, YTHDF2, YTHDF3, YTHDC1, and YTHDC2 mRNA levels are dramatically reduced in PTB patients infected with Mycobacterium tuberculosis and are negatively correlated with disease‐related clinical indicators such as erythrocyte sedimentation rate and alanine aminotransfer, indicating the critical role of m^6^A “reader” in PTB.^190^ The above findings indicate that m^6^A modifications are widely implicated in the pulmonary virus infection cycle, and targeting m^6^A can contribute to pioneering new antiviral treatment modalities. However, the exact mechanism of m^6^A modifications affecting the host's antiviral response is not fully understood.

## 4 TECHNIQUES FOR STUDYING RNA MODIFICATIONS IN PDs

### 4.1 RNA modification database

RNA modification database is a key component of RNA modification research. The use of these computer tools can contribute to a deeper understanding of the relationship between RNA modifications and PDs. With the gradual advancement of RNA modification research, a plentiful of databases with different functions have emerged in addition to the widely used NCBI‐GEO database, providing a foundation for advanced inquiry into the functions of RNA modifications. There are three primary classifications for existing RNA modification databases: biochemical RNA modification, reversible RNA modification, and RNA editing databases.^191^ The predominant repositories for biochemical RNA modifications are: RNA Modification Database and Modomics, which provide comprehensive information on the biochemical characteristics and biosynthetic pathways of RNA modifications.^192^, ^193^ Within the realm of reversible RNA modification databases, a distinction can be made between comprehensive databases and specialized databases, both instrumental in examining the multifaceted functional diversity and underlying mechanisms of RNA modifications within intricate regulatory networks. RMBase is unparalleled in its comprehensiveness as an RNA Modification Base Database,^194^ and other comprehensive databases include Methy‐Transcriptome Database,^194^, ^195^, ^196^ m^6^A‐Atlas,^196^ m^7^GHub,^197^ RNA Epitranscriptome Collection,^198^ m^5^C‐Atlas,^199^ and RM2Target.^200^ The design of specialized reversible RNA modification databases is more targeted than that of comprehensive databases, including CVm^6^A,^201^ RMVar,^202^ and RMDisease.^203^ Due to the fact that RNA editing is an irreversible posttranscriptional modification process, the databases related to RNA editing are isolated, including RNA Editing Database,^204^ Rigorously Annotated Database of A‐to‐I RNA Editing,^205^ Database of RNA Editing,^206^ REDIportal,^207^ and DirectRMDB.^208^ Leveraging these dynamically updated databases, we can sift through gene mutations linked to RNA modifications in PDs, facilitating the formation of disease‐relevant risk characteristics for evaluating disease prognosis or potential biomarkers.^209^, ^210^ In summary, multiple RNA identification methods can facilitate quick detection of RNA modification abnormalities in PDs and provide effective tools for accurate diagnosis and biomarker development.

### 4.2 Identification, quantification, and function of RNA modifications

The emergence of RNA modifications has broadened our understanding of gene regulation, leading to a resurgence of research activity in the field of RNA epigenetics. The development of RNA modification detection methods has allowed more and more RNA modifications to be identified.^211^ High‐throughput sequencing methods that identify RNA modifications mostly rely on specific antibodies to enrich RNA containing modification sites and recognize modifications at the RNA level,^71^, ^212^ such as PA‐m^6^A‐Seq and MeRIP‐Seq for m^6^A modification identification,^52^, ^213^ m^1^A‐ID‐Seq, m1A‐Seq, m^1^A‐MAP, and m^1^A‐Seq‐TGIRT for m^1^A identification,^214^, ^215^, ^216^ m^7^G‐MeRIP‐Seq and m^7^G miCLIP‐Seq for m^7^G identification,^96^, ^217^ as well as Bisulfite sequencing (BS‐Seq) for m^5^C identification.^218^

Another antibody‐independent sequencing method is based on chemical labeling, such as m^6^A‐label‐seq and m^6^A‐SEAL for identifying m^6^A modifications,^219^, ^220^ m^7^G‐Seq for identifying m^7^G modifications,^96^ N^3^‐CMC‐enriched pseudouridine sequencing (CeU‐Seq), Pseudouridine sequencing (Pseudo‐seq), and DM‐Ψ‐Seq for identifying Ψ sites.^92^, ^221^, ^222^ Alternative methods for detecting m^6^A modifications include deamination adjacent to RNA modification targets sequencing. Through the utilization of a fused APOBEC1‐YTH protein, this technique induces C‐to‐U editing in close proximity to the m^6^A site, enabling the identification of modification sites.^223^ Direct sequencing of single RNA molecules is made possible by next‐generation sequencing, obviating the requirement for conversion into DNA and thereby averting the loss of modified nucleotides. Regardless, the methodology's potential in discerning RNA modifications is highlighted, particularly in the case of m^6^A, which is distinguishable from nonmethylated adenine via SMRT sequencing.^224^ Through collective efforts, a range of methodologies are under development to scrutinize RNA modifications, paving the way for systematic exploration of the functional relevance of RNA modifications in both biological phenomena and human pathologies.

As the state of RNA alters with the growth and development of organisms, the modification levels of various RNAs also change accordingly. Thus, the assessment and quantification of RNA modifications can facilitate a deeper insight into distinct cellular regulatory processes. With the distinct physical and chemical attributes of modified and unmodified nucleotides in mind, employing liquid chromatography mass spectrometry (LC–MS) alongside single nucleotide digestion and ultraviolet detection provides a means to evaluate the comprehensive RNA modification level and undertake conventional quantification.^225^, ^226^ Traditional or two‐dimensional thin‐layer chromatography (TLC) can be used for qualitative analysis of RNA modifications, comparison of migration rates between samples and corresponding controls, and quantitative measurement of spot strength.^227^, ^228^ The binding of TLC and radioactive isotopes can further improve detection sensitivity, making it suitable for detecting low‐abundance RNA modifications.^229^

RNA immunoprecipitation (RIP) is mainly utilized to investigate RNA–protein interactions, usually in combination with high‐throughput sequencing methods such as m^6^A‐MeRIP‐seq, m^6^A‐ChIP‐seq, and Borohydride Reduction sequencing,^52^, ^98^, ^230^, ^231^ and is also commonly used to observe the involvement of RNA modifications in pathological processes of diseases.^160^ CRISPR is an RNA modification editing tool that can quickly and flexibly screen genomes including animals, plants, and microorganisms, achieve accurate recognition of modification sites, and conduct functional research on specific modification risk sites.^232^, ^233^ Conclusively, various RNA identification methods can encourage the quick detection of abnormal RNA modifications in PDs, which provide effective tools for accurate diagnosis and biomarker development.

## 5 THERAPEUTIC POTENTIAL

### 5.1 Lung cancer

Due to their essential role in the genesis and evolution of PDs, RNA modifications open up new avenues for early PD diagnosis and therapeutic approaches. Multiple investigations have validated that the aberrant regulation of RNA modifications or RNA‐modifying enzymes is a contributing factor to pulmonary functional disorders and lung cancer. As the research progresses in this field, numerous RNA‐modifying proteins have been reported as potential targets for cancer therapy.^130^ One example is miR‐338‐5p, which acts as a negative modulator of METTL3, suppressing the methylation modification of oncogenes and consequently impeding the proliferation of lung cancer cells. Additionally, miR‐33a directly targets the 3′‐UTR of METTL3 mRNA, leading to decreased METTL3 expression and further inhibition of NSCLC cell proliferation.^234^ Depletion of METTL3 expression can further impede tumorigenicity and heighten the sensitivity of lung cancer cells to BRD4 inhibition, implicating METTL3 as a viable candidate for cancer therapeutics.^123^ Furthermore, RNA modifications are advantageous in cooperation with drugs, which increase the clinical benefits of patients. An example of this is how m^6^A modifications can mitigate gefitinib resistance in NSCLC patients via the FTO/YTHDF2/ABCC10 axis.^235^ Depletion of YTHDF1 renters benefits to overcome cisplatin resistance in cancerous NSCLC cells through the Keap1–Nrf2–AKR1C1 axis.^138^ Moreover, the regulatory effect of the m^6^A epigenome on RNA homeostasis is also a mechanism for controlling drug resistance in cancer patients.^236^ Targeting YTHDF1, an m^6^A reader, shows considerable potential for enhancing the effectiveness of PD‐L1 immune‐checkpoint blockade in treatment.^237^ Lowered ADAR1 expression, responsible for A‐to‐I editing, heightens the susceptibility of cancer patients to immune‐checkpoint blockade.^153^ The overexpression of IL‐37, an m^6^A modification‐associated inflammatory cytokine, can depress A546 cell proliferation by modulating the level of m^6^A methylation and the expression of related molecules, which is beneficial for NSCLC patients.^238^ FTO, being an indispensable oncogene, plays a critical role in cancer progression. Inhibitors like FB23 and FB23‐2 exert their effects by directly targeting FTO, leading to inhibition of FTO demethylase activity. Consequently, this inhibition serves to attenuate cancer cell proliferation while promoting apoptosis.^239^ In parallel, R‐2HG, a separate inhibitor of FTO, heightens the responsiveness of tumor cells to therapeutic drugs, leading to tumor cell elimination.^240^ The competitive binding of Rhein to the catalytic domain of FTO results in robust inhibition of m^6^A demethylation, potentially triggering apoptosis and cell cycle arrest in NSCLC cells.^241^, ^242^ Altogether, it is of immense promise to combine traditional chemotherapeutic or immunomodulating drugs with RNA modification regulators for cancer treatment.

### 5.2 Other PDs

So far, research focuses on RNA modification targeted lung cancer therapy, other PDs are expected to have similar effects. Despite the initial stage of development, inhibitors aimed at mA methylation regulators are showing promise. More evidence is emerging to support the therapeutic role of RNA modification in treating various pulmonary disorders. LRPPRC and FTO, newly characterized m^6^A regulators, emerge as prognostic genes in IPF patients, indicating a favorable prognosis. Research further virtually screens out 10 compounds as potential drugs for targeting LRPPRC and FTO, suggesting these two regulators as therapy targets.^243^ Furthermore, suppressing METTL3 activity demonstrates the ability to restore proper differentiation of lung‐resident mesenchymal stem cells, preventing their aberrant transformation into myofibroblasts.^244^ In a comparable manner, the knockdown of ALKBH5 in vitro displays antifibrotic effects, suggesting novel therapeutic modalities for the treatment of IPF. However, within childhood allergic asthma patients, the absence of METTL3 in myeloid cells exacerbates allergic airway inflammation by promoting M2 macrophage activation, which proves counterproductive to disease alleviation efforts.^245^ Conversely, YTHDF1 fosters the proliferation and migration of airway smooth muscle cells in an m^6^A‐dependent manner, which supports asthma‐related airway remodeling. Despite its negative impact on disease alleviation, this mechanism offers a new avenue for therapeutic intervention in asthma.^246^ The process of FTO demethylation serves to stabilize FOXJ1 mRNA, promoting the development of motile cilia and subsequently suppressing the occurrence and advancement of asthma.^183^ Further research is required to confirm the therapeutic effect of RNA modification intervention on asthma. Moreover, numerous scoring methodologies integrating multiple m^6^A regulatory factors have been implemented in the care of lung cancer, IPF, and viral PDs, resulting in favorable outcomes for disease detection, risk stratification, and the evaluation of therapeutic efficacy and prognosis. Collectively, targeting RNA modification still has huge advantages in the treatment of PDs.

## 6 CONCLUSIONS AND FUTURE PERSPECTIVES

This review summarizes the execution processes dependent on RNA modifying enzymes (such as erasers, writers, and readers) of several important RNA modifications involved in PDs in both coding and ncRNAs, illustrates their impact on RNA processing, nuclear output, and RNA translation to decay, and further systematically analyzes the regulatory roles and targeted potential of these RNA modification regulators in the diagnosis and therapeutic interventions for PDs, including lung cancer, COPD, IPF, and lung injury caused by pathogen infection. In lung cancer, RNA modifications, especially METTL3‐mediated m^6^A modifications, seem to exert a greater impact on tumor proliferation, metastasis, and metabolism than other RNA modifications. Within IPF and pulmonary fibrosis, RNA modifications contribute to the onset and progression of these conditions by targeting disease‐associated fibroblasts and inflammatory pathways. Hence, these RNA modifications and associated regulatory proteins offer considerable potential as innovative diagnostic biomarkers and therapeutic targets for the early detection, management, and prognosis of PDs. Furthermore, the evolution of computer technology has made substantial contributions to the progress of research into RNA modifications, furnishing essential datasets for the analysis of factors associated with PDs. Therefore, we also briefly describe RNA modification‐related databases and identification tools crucial for promoting RNA epigenetics as a novel diagnostic biomarker with clinical value, enhancing the informativeness and practicality of this review.

Currently, research on RNA modifications is rapidly developing. Many unresolved matters persist in fully grasping the ramifications of RNA modifications in alleviating diseases. First, the advancement of various tools for RNA modification identification has revealed a plethora of over 170 different types of modifications. However, the presence of potential undiscovered modifications persists, and the precise mechanisms underlying these chemical alterations in both coding and noncoding RNAs remain unclear. RNA modifications may exert effects on nearly every RNA type, and their disruption of typical daily functions merits additional study. The extent to which RNA modifications regulate disease progression is also unclear. Second, writers, erasers, and readers constitute the fundamental regulators of RNA modifications and their functionality. Despite extensive research efforts, unraveling the mechanisms responsible for the dysregulated expression of these regulatory factors has proven elusive. Thirdly, the subject of m^6^A remains highly relevant in this field, owing to its extensive background knowledge and the utilization of mature research techniques. In the future, we shall focus more on other target modifications, perhaps providing more surprises in disease research. It is worth highlighting that apart from their function in maintaining RNA stability, m^6^A modifications also paradoxically act as a signal initiating RNA degradation.^38^ This mechanism is critical for dynamically regulating gene expression. Moreover, m^6^A‐mediated translational regulation is complex, which may either hinder translation or promote protein synthesis, depending on the specific RNA‐binding protein and cellular environment.^247^ Therefore, when m^6^A is considered a therapeutic target, the complexity of its biological effects should be emphasized. Finally, in the context of viral infection, since RNA modifications, especially m^6^A modifications, can support viral replication, we also need to fully consider the effect of RNA modifications on immune cells and complex immune networks, which contributes to a deeper understanding of PDs caused by respiratory virus infection. RNA modifications implicated in antiviral immunity may also possess broad research prospects. However, concerning the therapeutic advantages of these modifications, research into their functions within coding and noncoding RNAs remains at a preliminary stage, indicating the need for future studies to bridge these knowledge deficiencies. In addition, comprehensive and accurate characterization of RNA modifications can be achieved by combining multiple complementary technologies and methods, and novel and advanced tools shall be utilized to establish a definite association between RNA epigenetics and PDs. Treatment strategies targeting RNA modifications for PDs are primarily in the conceptual phase, highlighting the need for extensive clinical trials to evaluate the diagnostic and therapeutic implications of RNA modifications in PDs.

## AUTHOR CONTRIBUTIONS

W. Q. provided ideas and outlines, L. Y. collected messages and references, W. Q. and T. L. prepared original manuscript, L. Y. and T. L. designed images and tables, and W. L. revised manuscript and polished the language. W. Q., J. Z., and S. X. were responsible for review, proofread, and editing. The article was a result of joint effort from all authors, who have all given their approval for the submitted version.

## CONFLICT OF INTEREST STATEMENT

The authors declare that they have no conflict of interest.

## ETHICS APPROVAL

Not applicable.

## Data Availability

Not applicable.
